# The impact of air travel on the precocity and severity of COVID-19 deaths in sub-national areas across 45 countries

**DOI:** 10.1038/s41598-022-20263-y

**Published:** 2022-10-03

**Authors:** Ettore Recchi, Alessandro Ferrara, Alejandra Rodriguez Sanchez, Emanuel Deutschmann, Lorenzo Gabrielli, Stefano Iacus, Luca Bastiani, Spyridon Spyratos, Michele Vespe

**Affiliations:** 1 Sciences Po, Centre for Research On Social Inequalities (CRIS), CNRS, Paris, France; 2 Migration Policy Centre (MPC), European University Institute, Florence, Italy; 3 European University Institute, Florence, Italy; 4 Humboldt Universität, Berlin, Germany; 5 Deutsche Zentrum für Integrations-und Migrationsforschung (DeZIM), Berlin, Germany; 6 Europa-Universität Flensburg, Flensburg, Germany; 7 European Commission, Joint Research Centre (JRC), Ispra, Italy; 8 Institute for Quantitative Social Science, Harvard University, Cambridge, MA USA; 9 Institute of Clinical Physiology, Consiglio Nazionale delle Ricerche (CNR), Pisa, Italy

**Keywords:** Diseases, Risk factors

## Abstract

Human travel fed the worldwide spread of COVID-19, but it remains unclear whether the volume of incoming air passengers and the centrality of airports in the global airline network made some regions more vulnerable to earlier and higher mortality. We assess whether the *precocity* and *severity* of COVID-19 deaths were contingent on these measures of air travel intensity, adjusting for differences in local non-pharmaceutical interventions and pre-pandemic structural characteristics of 502 sub-national areas on five continents in April–October 2020. Ordinary least squares (OLS) models of *precocity* (i.e., the timing of the 1st and 10th death outbreaks) reveal that neither airport centrality nor the volume of incoming passengers are impactful once we consider pre-pandemic demographic characteristics of the areas. We assess *severity* (i.e., the weekly death incidence of COVID-19) through the estimation of a generalized linear mixed model, employing a negative binomial link function. Results suggest that COVID-19 death incidence was insensitive to airport centrality, with no substantial changes over time. Higher air passenger volume tends to coincide with more COVID-19 deaths, but this relation weakened as the pandemic proceeded. Different models prove that either the lack of airports in a region or total travel bans did reduce mortality significantly. We conclude that COVID-19 importation through air travel followed a ‘travel as spark’ principle, whereby the absence of air travel reduced epidemic risk drastically. However, once some travel occurred, its impact on the severity of the pandemic was only in part associated with the number of incoming passengers, and not at all with the position of airports in the global network of airline connections.

## Introduction

That ‘travel is a potent force in disease emergence and spread’ is a mainstay of public health research^1^. Epidemics are multi-scale network phenomena that hinge on human mobility as a source of contacts across social contexts^2^. In a cascade-like process, *long-distance travel* is held to initiate local outbreaks. Consequently, *population mixing* propagates the disease locally. Differences in demographic and health-related *predispositions of the local population* eventually lead to a higher or lower incidence of the disease.

There have been studies investigating the influence of these three individual factors—long-distance travel, population mixing, and local predispositions—on the spread of COVID-19, but rarely have such factors been considered simultaneously^3,4^. While our primary focus is on the role of the first factor—long-distance travel, which diminished but did not halt in 2020—we also adjust for the two other factors through numerous covariates (related to the demographic and public health characteristics of the areas) to account for differences in timing and impact of the disease across localities.

We assess whether the *precocity* (or timing) and the *severity* (or incidence) of the COVID-19 pandemic in 2020 was contingent on the volume of airline passenger inflows, the position of airports in the global network of commercial flights, and travel limitation policies. We do so *dynamically* with weekly data and taking *sub-national areas* as our research units. This allows us to study the effect of airline travel at a higher granularity than the national level examined by most existing studies. At the same time, this granularity makes our models more inclusive of local variations in non-travel related drivers of the pandemic. Such variations may be especially important in larger and more diverse countries, where the national average can overshadow diverse area risks, which is sometimes highlighted as a serious limitation of existing comparative studies^5^.

Well before the COVID-19 pandemic, research delved into modelling the relationship between the characteristics of the global travel network—relying mostly on airline travel, as we do in this paper—and the timeline of epidemics’ spread^6^. These studies have leveraged the inherent network properties of transportation infrastructures, which exemplify wider interconnected socio-technical systems^7^ that are independent of specific diseases and other possible human and environmental covariates^8^. Other studies have compared the effects of international routes and local mobility parameters, in particular commuting, showing that the latter has a stronger impact on the spread of epidemics in the periphery of the global travel network^9^. Similarly, several studies prove that limits to personal interactions at the local level curb overall epidemic prevalence more than reduced long-haul travel does, unless travel restrictions are timely and drastic^10–12^. An analysis of the effect of pre-existing levels of international travel on the evolution of the 1889, 1918, 1957 and 2009 pandemics found that even extremely large historical variations in global mobility (from the late nineteenth century to the early twenty-first century) affected the speed of cross-country transmission of pandemics only minimally^13^. The same study shows that final mortality rates by country are statistically independent from the time of the outbreaks in these historical pandemics.

For the COVID-19 pandemic, it is ascertained that its outbreak was fed by human travel in all nations examined outside China^14–16^. In particular, the disease was introduced by air passengers in 71 percent of the first registered country cases worldwide^17^. While abundant^18^, however, research on the relationship between air travel and the COVID-19 pandemic dynamics has been mostly simulation-based, aiming to perform scenario analyses and forecasts. Observational studies like ours, which aim at assessing the impact of airline travel on the *precocity* and the *severity* of the pandemic, are scarce.

As for *precocity*, epidemic spreading models found that airline connections worked well to predict the timing of outbreaks in different areas of China^19^. Potential exposure to the virus due to airline networks outside China was identified early on^20^, but the impact of flight connectivity on the pandemic’s onset has only been assessed through simulations which concluded that limiting air traffic could, at most, ‘buy time’ in the early phase of the pandemic^21–25^. Although seminal and useful for rapid policy responses, these nowcasting ‘studies (had to) make assumptions which are independent of the specifics of COVID-19; and accordingly, their findings have to be taken with caution when applying them to the spread of COVID-19’^18^. No observational study, to the best of our knowledge, has sought to unravel the effect of flight connectivity, air passenger volume and travel restrictions on the timing of the varying outbreak of COVID-19 in different parts of the world retrospectively.

As for the *severity* of the pandemic, there is a vast literature claiming that higher exposure to air passenger inflows (both from China and elsewhere) and a later introduction of travel restrictions provoked a stronger incidence of the virus^26–32^ and also a higher mortality^33^. However, other studies highlight that in May 2020 imported cases accounted for no more than 1 to 10 percent of total cases in two thirds of countries^34^. The bulk of contagions occurred through local contacts and a study of 184 countries shows that in the first semester of 2020 local mobility restrictions were more effective in reducing COVID-19 incidence than international travel limitations^35^.

A general problem is that results relying on the number of COVID-19 *cases* must be taken with a grain of salt, as case detection—especially in the early stages of an epidemic—is highly questionable, depending on the availability of tests and the propensity of the population to be screened, which varied considerably from one country to another. On average, by March 2020 no more than a quarter of global COVID-19 infections were detected by surveillance systems^36^. For this reason, we opt for using data on the number of COVID-19 *deaths* per week as our key dependent variable when assessing the relationship between air travel and the precocity and severity of the pandemic in the following (models using cases are shown in Supplementary Information).

Drawing on the most prevalent findings of the literature, we formulate and test three hypotheses. The first hypothesis is about the *precocity* of COVID-19 outbreaks:

### (H1)

Areas with larger airline travel volume and higher airport centrality experienced earlier outbreaks of COVID-19 deaths (the ‘travel as timer’ hypothesis).

The second and third hypotheses refer to the *severity* of COVID-19 impact:

### (H2)

Areas with larger airline travel volume and higher airport centrality experienced a stronger incidence of COVID-19 deaths, and even more so in the case of travel originating in more infected areas (the ‘travel as fuel’ hypothesis).

### (H3)

With importation progressively contained by travel limitation policies, air travel volume and airport centrality had a diminishing importance on the incidence of COVID-19 deaths (the ‘travel as spark’ hypothesis).

Our timeframe is April–October 2020—the period between the WHO’s declaration of the COVID-19 pandemic and the completion of the first wave in most parts of the planet (https://www.ecdc.europa.eu/en/geographical-distribution-2019-ncov-cases). Our findings align with the ‘travel as timer’ hypothesis (H1) to a limited extent, as the centrality of destination airports in the global network of flights is not a significant predictor of earlier outbreaks, and the volume of air passengers is insignificant once we control for local demographic characteristics. The ‘travel as fuel’ hypothesis (H2) is also not corroborated but rather qualified, with the number of incoming passengers having an impact, especially from more infected areas, but not airport centrality. The inhibiting effect of travel bans or the absence of airports altogether is also highlighted. Lastly, the decline of the effects of air travel volume on COVID-19 death incidence over time confirms the ‘travel as spark’ hypothesis (H3).

## Data and methods

The analyses presented in this article are based on the novel *Sub-National COVID-19 Incidence and Determinants Dataset*, which includes 502 sub-national areas of 45 countries in five continents (Europe, Asia, North America, South America and Oceania). The information used to build this dataset was drawn from a variety of sources in order to cover four major areas of interest: health outcomes of the pandemic (COVID-19 cases and deaths), air travel variables (number of incoming air passengers, centrality of local airports in the global airline network, air travel limitation policies), population mixing and non-pharmaceutical interventions (NPIs, based on the Oxford-tracker stringency index), and pre-pandemic area characteristics (demographics, public health and prior mortality rates). Details on the sources and the variables can be found in the accompanying document of the dataset. All data come from public sources, with the exception of information on air passenger volume, which we were authorized to draw from Sabre Travel Data, a private company dataset on airport traffic collected directly from the airline industry (https://www.sabre.com). These data were further processed and aggregated by areas in our dataset. In this article we model the *precocity* (i.e., the calendar week of the 1st and 10th COVID-19 deaths) and *severity* (i.e., the weekly COVID-19 number of deaths) of COVID-19 through ordinary least squares (OLS) and negative binomial mixed (i.e., GLMM with negative binomial link function) models. We draw on a plethora of model specifications adjusting for variables that were found to affect the outcome significantly in previous studies (Table 1).

**Table 1:** Description of the variables used in the models of precocity and severity of COVID-19 (April–October 2020). *Source*: Sub-National COVID-19 Incidence and Determinants Dataset.

Variable	Description	Spatial scope*	Time scope
Weekly deaths (dependent variable)	COVID-19 related weekly deaths	Subnational	Weekly
**International mobility**			
Incoming passengers	Total incoming airline passengers in NUTS2 region, lagged by 1 month	Subnational	Monthly
Importation risk	COVID-19 related weekly deaths per capita in NUTS2 sending regions weighted by share of total incoming passengers from each region, lagged by 1 month	Subnational	Monthly
Airport centrality	Eigenvector centrality of airports in international travel network, where edges are weighted by number of passengers (median of airports in region), lagged by 1 month	Subnational	Monthly
Absence of airports	No airport in the region (dummy variable)	Subnational	Time-invariant
**Travel restrictions**			
None/minimal	No travel ban or only screening and/or quarantining at arrival, lagged by 3 weeks	National	Weekly
Partial bans	Ban on arrivals from certain regions, lagged by 3 weeks	National	Weekly
Total ban	Ban on all regions or total border closure, lagged by 3 weeks	National	Weekly
**Local factors**			
Population	Size of resident population	Subnational	Time-invariant
Oxford-tracker stringency index (modified)	Index summarizing containment and closure policies including: school closure, workplace closing, cancelling of public events, restrictions on gatherings, closing of public transport, stay at home requirements and restrictions on internal movement; not containing air travel policies, lagged by 3 weeks	National	Weekly
Real GDP pc PPP	Real GDP per capita PPP at constant prices (millions of USD)	Subnational	Time-invariant
Population density	Population per km^2^	Subnational	Time-invariant
Hospital beds per 1000	Hospital beds per 1000 inhabitants	National	Time-invariant
Share of 65 +	Share of population of age 65 or over (%)	Subnational	Time-invariant
Cardiovascular death rate	Age-standardized deaths from cardiovascular diseases per 100 k inhabitants	National	Time-invariant
Cancer death rate	Age-standardized deaths from cancer per 100 k inhabitants	National	Time-invariant
Prevalence of adult obesity	Percentage of adults aged 18 and over with Body Mass Index (BMI) of 30 kg/m^2^ or higher	National	Time-invariant
Week of first COVID death	Week of the year in which first COVID-related death was recorded	Subnational	Time-invariant

*The subnational level corresponds to the NUTS2 level in Europe and comparable administrative regions in non-European countries (e.g., in the US corresponding to Federal States).

Research on between-country variations in COVID-19 severity has consistently highlighted the effect of several pre-existing comorbidities including a senior population structure, a higher GDP per capita and a lower number of hospital beds^37,38^. The impact of population density, obesity and air pollution is empirically less clear, with some within-country ecological studies disproving their independent impact on COVID-19 mortality^39,40^. We include these potential predictors in the following analyses as *Structural predispositions*, with the only exception of air pollution, for which we have 20 percent missing cases (analyses including this variable are in Supplementary Information). There is also strong evidence that everyday human interactions are highly conducive to the spread of COVID-19—and their reduction conversely reduces contagion^5,41–43^. We operationalize such *Population mixing* through the ‘Oxford-tracker modified stringency index’ of NPIs, lagged by three weeks^44^. Given that basically all forms of NPIs aim at a reduction of human interactions, we deem this single indicator to be the most parsimonious way of tapping changes in population mixing over time. This is also in light of research based on mobile positioning data that indicates overall compliance with such measures in the first semester of 2020^45^. The index is modified by removing its international travel limitation component, since travel limitation policies are included among our covariates separately.

Our core variables revolve around *Long-distance mobility*: air passenger volume per capita (lagged by one month), the average incidence in the sending regions of these passengers (or ‘importation risk’, lagged by one month), the centrality of the subnational area in the global network of commercial flights (see Table 1 for the operational definition; lagged by one month), the absence of airports in the region, and the air travel restriction policy that was in place three weeks earlier. Air travel restriction policies are operationalized in three categories: total travel ban as a baseline, travel bans for some routes, and less disruptive limitations (like temperature-screening, quarantines for incoming passengers, or no limitations at all). Lags of three weeks are introduced assuming, from existing literature, an average COVID-19 infection-to-death delay of 20–23 days^46,47^. We extended the lag to one month for our travel indicators given their monthly nature. Finally, we introduced a variable that accounts for *Recursive effects*—namely the calendar week of the first COVID-19 death (which is the dependent variable in the analysis of ‘precocity’, Table 2). This factor incorporates what has been called the ‘surprise effect’ of COVID-19, as an earlier outbreak could bring about a less efficient public health response^48^. Descriptive statistics for each of these variables are in the Supplementary Information. To ensure comparability of the estimated coefficients across covariates and across time, all variables were standardized in our analyses.

**Table 2:** The impact of late 2019 air passenger traffic on the precocity of COVID-19 outbreaks (calendar week of occurrence of 1st and 10th death) [2A: traffic from Chinese airports; 2B: traffic from other airports]. *Source*: Sub-National COVID-19 Incidence and Determinants Dataset.

DV: Calendar week of 1st or 10th COVID-19 death	(1)	(2)	(3)	(4)	(5)	(6)	(7)	(8)
	Wk 1st	Wk 1st	Wk 1st	Wk 1st	Wk 10th	Wk 10th	Wk 10th	Wk 10th
**2A: Airline passengers from Chinese airports**								
No airport in region	0.122 (0.398)	− 0.239 (0.391)	0.343 (0.345)	− 0.044 (0.333)	2.051* (0.810)	0.975 (0.764)	1.903** (0.697)	1.378* (0.677)
Inbound passengers (end 2019)	− 1.130*** (0.269)	− 0.049 (0.332)	− 0.489 (0.307)	− 0.199 (0.297)	− 1.592** (0.536)	0.864 (0.633)	− 0.112 (0.627)	− 0.439 (0.609)
Airport centrality (end 2019)	− 0.442 (0.248)	− 0.346 (0.261)	0.026 (0.230)	− 0.041 (0.219)	− 0.840 (0.494)	− 0.346 (0.497)	0.102 (0.454)	− 0.100 (0.434)
Population		− 2.730*** (0.608)	− 2.610*** (0.580)	− 2.611*** (0.554)		− 5.127*** (1.172)	− 4.146*** (1.158)	− 4.327*** (1.113)
Population density (pop/km2)		− 0.280 (0.207)	− 0.201 (0.181)	− 0.304 (0.173)		− 0.640 (0.395)	− 0.659 (0.356)	− 0.812* (0.345)
Real GDP pc PPP		− 0.413** (0.136)	0.041 (0.130)	0.173 (0.131)		− 1.874*** (0.294)	− 0.483 (0.305)	− 0.372 (0.315)
Hospital beds per 1000 residents				0.207 (0.249)				1.847*** (0.493)
Share of 65 +				-0.699*** (0.188)				-1.512*** (0.390)
Cardiovascular death rate				0.901*** (0.249)				− 0.115 (0.514)
Cancer death rate				0.313 (0.186)				1.141** (0.379)
Prevalence of adult obesity				− 0.430 (0.354)				− 2.071** (0.718)
Observations	453	453	453	453	429	429	429	429
R-squared	0.056	0.122	0.345	0.423	0.054	0.192	0.359	0.429
continent fixed effects	No	No	Yes	Yes	No	No	Yes	Yes
**2B: Airline passengers from non-Chinese airports**								
No airport in region	− 0.203 (0.393)	− 0.307 (0.389)	0.216 (0.341)	− 0.103 (0.329)	1.341 (0.793)	0.947 (0.763)	1.767* (0.690)	1.256 (0.667)
Inbound passengers (end 2019)	− 1.229*** (0.192)	− 0.439 (0.268)	− 0.406 (0.236)	− 0.421 (0.234)	− 2.435*** (0.381)	− 0.345 (0.530)	− 0.451 (0.480)	0.138 (0.476)
Airport centrality (end 2019)	− 0.120 (0.148)	0.042 (0.155)	0.114 (0.138)	0.139 (0.132)	0.175 (0.290)	0.436 (0.297)	0.382 (0.272)	0.182 (0.262)
Population		− 2.664*** (0.735)	− 3.491*** (0.660)	− 2.773*** (0.679)		− 4.602** (1.422)	− 5.006*** (1.312)	− 7.148*** (1.359)
Population density (pop/km2)		− 0.285 (0.202)	− 0.167 (0.176)	− 0.266 (0.170)		− 0.661 (0.389)	− 0.624 (0.349)	− 0.864* (0.338)
Real GDP pc PPP		− 0.353* (0.145)	0.065 (0.135)	0.206 (0.135)		− 1.804*** (0.323)	− 0.414 (0.323)	− 0.515 (0.329)
Hospital beds per 1000 residents				0.225 (0.247)				1.946*** (0.490)
Share of 65 +				− 0.675*** (0.187)				− 1.459*** (0.388)
Cardiovascular death rate				0.834*** (0.247)				− 0.154 (0.510)
Cancer death rate				0.311 (0.183)				1.133** (0.373)
Prevalence of adult obesity				− 0.333 (0.357)				− 2.140** (0.720)
Observations	452	452	452	452	428	428	428	428
R-squared	0.110	0.144	0.366	0.440	0.116	0.193	0.367	0.439
continent fixed effects	No	No	Yes	Yes	No	No	Yes	Yes

OLS regressions without and with continent fixed effects.

Standard errors in parentheses. *p < 0.05, **p < 0.01, ***p < 0.001.

## Results

### Precocity of COVID-19 outbreaks

In this section, we test whether the precocity of the outbreaks of the pandemic could be predicted on the basis of airline passenger inflows from the hotbed of COVID-19 (mainland China) or through other air travel routes. In Table 2 we focus on the calendar week of occurrence of the first death in our sample, which ranges from 7 (second week of February 2020, in Southern Canto, Japan) to 44 (last week of October 2020, in Yukonk, Canada). We estimate models that include passenger volume from Chinese airports and from other world airports separately (Table 2A,B) to avoid collinearity issues since the two figures are quite correlated (R = 0.60). As a sensitivity check, we also repeat the analysis for the week of occurrence of the 10th COVID-19 death (Models 5–8)^33^. Negative coefficients indicate an earlier outbreak.

A preliminary analysis showed no effect at all for airport centrality and a significant effect of the volume of incoming passengers from flights originating from China and from other world airports (see Supplementary Information, Table B1). Upon closer inspection, however, we found that this result was driven by the case of India alone (see Fig. B2), where all regions had very few incoming travelers and close-to-zero (officially registered) deaths in the early stage of the pandemic, which has been subsequently found to be an extraordinary undercount^49^. In the following, we discuss estimates from models where Indian regions and four other outliers (two in Japan, two in the USA: see Fig. B1) were dropped. In these models, none of our air traffic indicators predict an earlier onset of COVID-19 mortality. Passenger volume is still *prima facie* impactful, but not once we take into account population size, which increases the likelihood of having a COVID-19 deadly outbreak before other areas, as well as population density (models 2, 3 and 7). Demographic and health characteristics—in particular, a larger senior population and a higher prevalence of obesity—amplify the likelihood of an earlier onset, further diminishing the impact of the volume of incoming passengers (models 4 and 8). The vulnerability of older and obese people was particularly acute at the first manifestations of COVID-19, when health system responses were struggling with the specificity of the virus. The effects of these factors are similar whether we estimate them in conjunction with the number of passengers from China or from the rest of the world. Interestingly, pre-pandemic cardiovascular and cancer death rates are inversely related to the precocity of the onset of COVID-19 deaths, possibly because areas with a higher incidence of these diseases are more prepared to tackle medical emergencies. A larger availability of hospital beds—which is likely to attest more broadly to the strength of the local health system—also counters an earlier spread up to the 10th recorded death.

With regards to H1 (the ‘travel as timer’ hypothesis), the risk of experiencing an earlier outbreak of COVID-19 deaths is not conditional on airport centrality. On balance, the volume of incoming passengers (from China and elsewhere) is less impactful than a larger, older, and overweight population.

The same analyses were repeated with the occurrence of the 1st and 10th COVID-19 *cases*—which are known to be less reliable indicators of the outbreak than death counts, due to different testing and recording efforts across countries. They show a stronger and significant effect of incoming passengers, which may reveal—on top of previous considerations on the differential reliability of case detection in different parts of the planet—the more direct influence of travel on the spread rather than the fatality of the epidemic (see Supplementary Information, Table B2). At the same time, the data suggest an earlier emergence of cases in areas with a larger senior and obese proportion of the population, possibly because these groups experienced more serious symptoms, which also exposed them to the previously discussed higher risk of mortality at the beginning of the epidemic.

### Modeling the severity of COVID-19 deaths in April–October 2020

**Figure 1:**
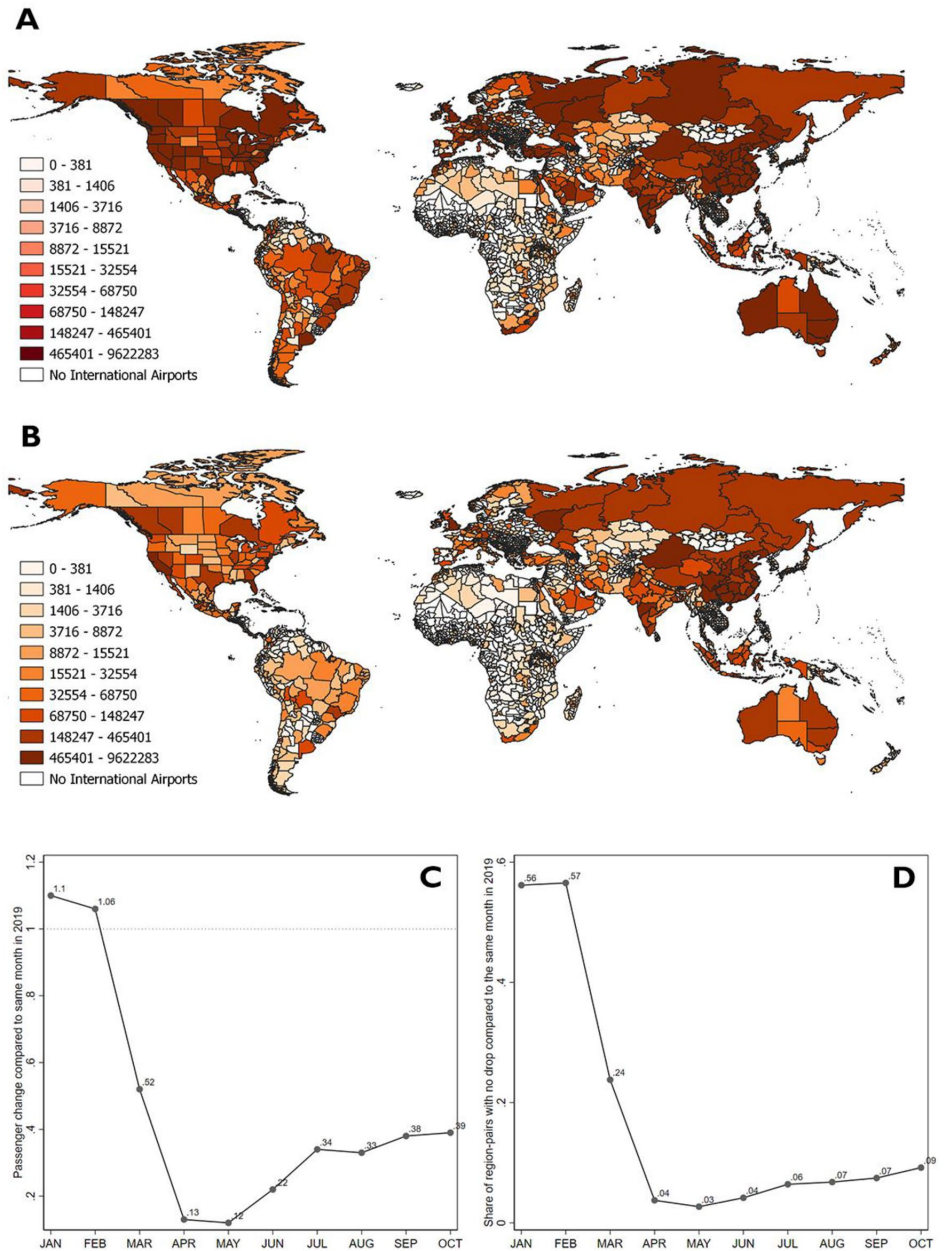
The drop in global air traffic induced by COVID-19. Panel (**A**) Shows the number of incoming passengers by subnational areas in April 2019; panel (**B**) shows the situation one year later in April 2020. Panel (**C**) shows the average change in the monthly number of air passengers in 2020 compared to 2019 (with a value smaller than 1 indicating a drop in passengers). Panel (**D**) shows the share of region-pairs that saw no drop in air passenger traffic in 2020 compared to the same month in 2019. A drop is defined here as the number of passengers reaching 90 per cent or less of the value in the previous year (assuming that a drop of less than 10 per cent is not meaningful and may simply indicate random fluctuation). *Source*: Sub-National COVID-19 Incidence and Determinants Dataset.

Although travel limitations were pervasive as a first defense against the spread of the new virus, air traffic did not completely stop in the wake of the pandemic. Our dataset includes information on the number of incoming passengers along all flight routes. The bulk of them diminished drastically after March 2020. Radical drops—with full cancellation of routes—affected Asia particularly (Fig. 1A [April 2019] and B [April 2020]). Globally, the decline in the volume of passengers was dramatic (Fig. 1C). At its lowest, in May 2020, the number of air passengers was 12 percent of the corresponding 2019 figure and, until October 2020, it had recovered to no more than 39 percent. In May 2020, only 3 percent of routes had not experienced any decline in air passengers compared to the same month one year earlier (Fig. 1D). However, air traffic was not entirely cancelled, as Fig. 1B shows. Some passengers continued to move between most parts of the globe. Potentially, this might have led to a higher incidence of the disease where larger numbers of incoming travelers continued to arrive, especially if their origin was from highly infected zones. Therefore, in the following we not only model the impact of the number of incoming air passengers on the receiving areas’ incidence of COVID-19 between April and October 2020, but also the interaction between this variable and the average incidence of the disease in the sending areas—that is, the COVID-19 importation risk.

### Weekly incidence: a negative binomial mixed model specification

In this section, we test H2 (the ‘travel as fuel’ hypothesis) on the impact of airline mobility by estimating a generalized mixed model (or multilevel model) which allows us to pool together all weekly information for the April–October 2020 period. Mixed models adjust for the nested data structure of our sub-national areas within countries, also taking into account the fact that we have longitudinal measures. In other words, each week for which we have data on deaths (level 1) is nested within each of the sub-national areas (level 2), which themselves are nested within countries (level 3) and within continents (level 4). By adjusting for time-based change, we can factor out heterogeneities in individual sub-national areas, as well as other existing heterogeneities between countries and continents^50^. Our specification also includes a time predictor (i.e., calendar week), as well as time-varying and time-invariant predictors. We fit a generalized linear mixed-effects model (GLMM) for the negative binomial family to our data with a random intercept and slope for the time indicator (calendar week). We assume a known overdispersion parameter of 1.2 (results do not vary much for other choices of the parameter; available upon request). One advantage of this approach is that it models changes over time at the local level simultaneously (e.g., how fast the pandemic evolves) and differences in time-wise change across different areas (e.g., differences in the peaks and valleys of the pandemic).

**Figure 2:**
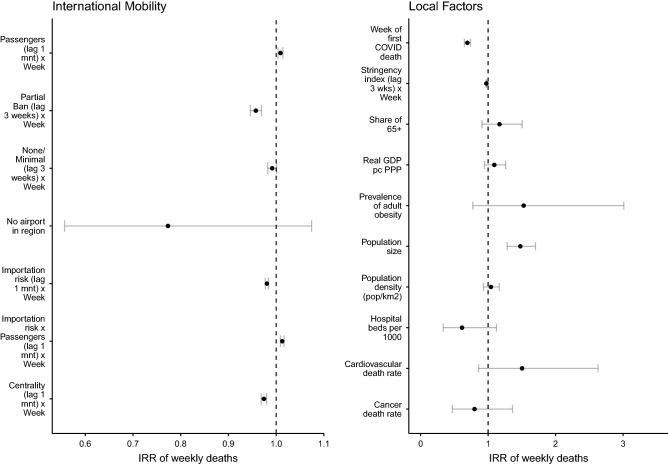
The impact of air passenger traffic on the severity of COVID-19 deaths (number of deaths per week) in April–October 2020, controlling for population mixing (NPI), structural predispositions and recursive effects. Generalized linear mixed-effects models. Risk ratios and confidence intervals. For full model specification with coefficients, see Table C1 in Supplementary Information. *Source*: Sub-National COVID-19 Incidence and Determinants Dataset.

Figure 2 reports the interaction between each of the dynamic variables (i.e., related to long-distance mobility and population mixing) and calendar week. The estimated risk ratios for long-distance mobility variables are slightly positive, with an increase of COVID-related death risk of about 1 to 5 per cent per one SD increase in the number of incoming passengers. This is not the case for airport centrality, however, which even entails a lower level of risk. The effect of travel limitation policies is complex and can be better spelled out by looking at models with a sequential inclusion of covariates (Supplementary Information). Table C1 (models 0–5) shows that partial or none/minimal bans on travel in general imply a higher number of deaths per week than total bans. In particular, areas that adopted the lowest level of travel restrictions experienced on average a 6 percent higher number of COVID-related deaths and areas which implemented partial travel bans faced a 1 percent higher number of deaths than areas which opted for total bans of air travel. However, once we adjust for the stringency of other NPIs (model 6), this is no longer the case. This last result speaks to the difficulty of establishing the direction of the effect of air travel bans. While infected individuals who travel by plane may spread the virus in the destination region, travel limitations can also affect the virus transmission in the area of origin by ‘trapping’ the virus within this same area.

We find a marked but imprecisely estimated association between the risk of COVID deaths and a radical policy of air travel limitation—namely, the absence of airports in the region (Fig. 2). The association is not significant given the small number of regions without an airport and its variation in different months (see next section). Structural predispositions—particularly, population size, cardiovascular death rate and obesity—have a greater bearing on the severity of the pandemic, though the effect of the last two factors is not statistically significant. A later outbreak and a stronger public health system (indicated by the number of hospital beds per residents) seem to limit the number of deaths, although the latter factor is not statistically significant. All other factors are *ceteris paribus* less impactful, including stringent NPIs put in place as a response to an already virulent pandemic, which probably counterbalance a reduction of severity with reverse causality. Finally, the random terms in our model indicate that most of the variation takes place at the subnational and country levels (Supplementary Information, Fig. C1).

### Weekly incidence: Modeling months separately

This section presents an alternative modeling strategy. Our dependent variable is still weekly incidence (COVID-19 deaths), but we concentrate on the first weeks of April, May, June, July, August, September and October 2020 separately. With this analysis we aim to check the robustness of the multilevel models discussed in the previous section while digging deeper into the time-specific impact of different factors during the first wave of the pandemic, thus testing H3 (the ‘travel as spark’ hypothesis).

**Figure 3:**
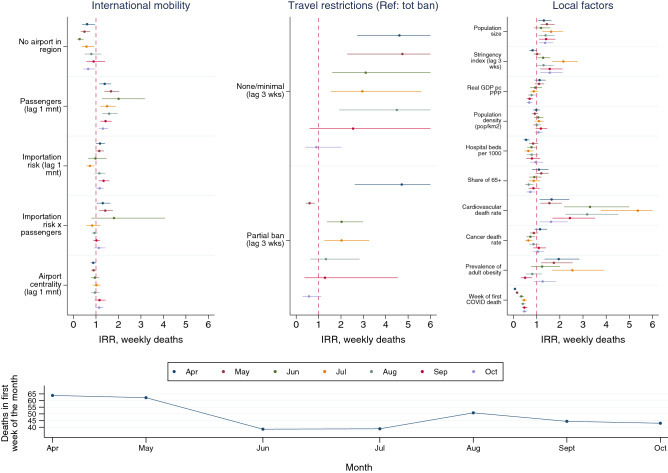
The impact of air passenger traffic on the severity of COVID-19 deaths (number of deaths in the first week of April–October 2020), controlling for population mixing (NPI), structural predispositions and recursive effects. Negative binomial regressions with continent fixed effects. Risk ratios and confidence intervals, For full model specifications with coefficients, see Table C2 in Supplementary Information. *Source*: Sub-National COVID-19 Incidence and Determinants Dataset.

Figure 3 reports the full models for the first week of each month. The severity of COVID-19 deaths in receiving areas was not significantly impacted by airport centrality at any time. The number of incoming passengers, and the risk associated with them coming from areas with higher incidence (measured through an interaction factor), did enhance the severity of the pandemic, particularly in the April-June period. In June, the effect of incoming passengers reaches its peak: a one SD increase in incoming airline travelers doubled COVID-19 deaths. Conversely, the absence of airports in the region is particularly protective against a more severe pandemic between April and June. Areas which imposed no or only minimal restrictions (such as screening passengers or quarantining them) fared definitively worse than areas that took a stricter approach. For example, in April and May, areas that opted for such policies—that is, did not adopt travel bans—experienced almost five times more COVID-related deaths than areas with total bans, *ceteris paribus*. In April, partial travel bans proved as ineffective as moderate travel policies. Full travel bans, in other words, were especially effective in the early stages of the pandemic, in line with previous studies^51^.

As for controls, their effect is not always as expected or significant. In particular, the lagged Oxford stringency index (modified in order to exclude travel policies, which we considered separately, as in the prior section) was found to associate negatively with the severity of the pandemic only from June onwards. A possible explanation is reverse causality: stricter measures against population mixing had to be taken in areas with rising COVID-19 mortality. More hospital beds predict a lower COVID-19 mortality at all stages, while population size and the precocity of the pandemic boost incidence. The cardiovascular mortality rate and population obesity are significant predictors until August, but their effect declines and even reverts for obesity at the end of our period of observation.

### Weekly incidence: modeling by epidemiological weeks

The previous analyses treated COVID-19 mortality by calendar weeks of the year 2020. In this section we repeat them aligning data by epidemiological week (i.e., the week since the first COVID-19 death) instead, thus following the evolution of the pandemic, regardless of the different timing whereby the virus spread across the world. We focus on the thirty weeks immediately after the outbreak to further test H3 on the changing impact of air travel over time.

**Figure 4:**
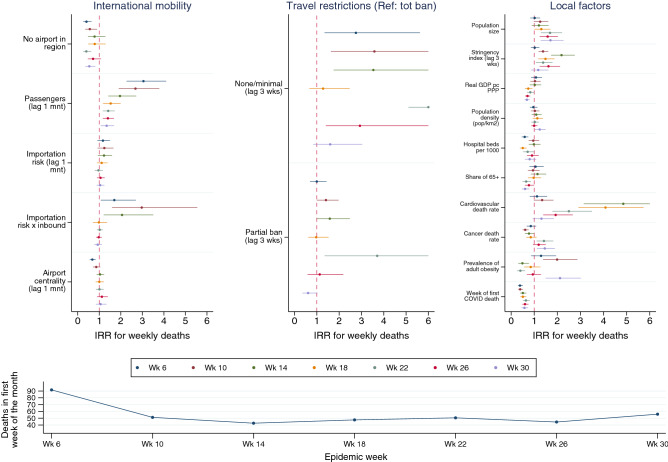
The impact of air passenger traffic on the severity of COVID-19 deaths in the first 30 epidemiological weeks (week1 = first COVID-19 death) controlling for population mixing (NPI), structural predispositions and recursive effects. Negative binomial regressions with fixed effects by continent. Risk ratios and confidence intervals. For full model specifications with coefficients, see Table C4 in Supplementary Information. *Source*: Sub-National COVID-19 Incidence and Determinants Dataset.

Figure 4 reports results for epidemiological weeks 6, 10, 14, 18, 22, 26 and 30. Compared to the previous one, which compared areas that were at different stages of the pandemic, this analysis better reveals the evolution of the effect of some factors over time.

The volume of incoming passengers has a significant but progressively declining impact on the number of COVID-related deaths, as hypothesized by H3 (the ‘travel as spark’ hypothesis). In weeks 6 to 14, the number of passengers had a larger impact on COVID deaths. This was particularly the case when passengers came from areas with higher COVID incidence (i.e., with stronger importation risk). This effect disappears with the passing of time, as we expected given the increasing controls on travelers’ health at departure. Overall, its magnitude remains modest compared to the effect of travel limitation policies. On average, these latter have a larger impact, but without a clear trend. The effects of structural factors are relatively stable, with the exception of the cardiovascular mortality rate, which boosts COVID-related deaths strongly in the first 14 weeks of the epidemic but declines markedly and gradually in the following. All other factors have an influence similar to the previous analysis and do not show any regular dynamic over time.

## Discussion

As an airborne disease, the spread of COVID-19 worldwide was facilitated by human travel. Most existing studies taking into account the travel-pandemic nexus have been concerned with the prediction of disease arrival and incidence in a nowcasting perspective. Such simulation studies are known to face major problems in the case of complex events like pandemics^52,53^. Instead, our approach was observational and retrospective, aiming to assess what happened in different areas of the world in response to their exposure to incoming air travel as well as specific combinations of pre-existing structural conditions and emerging policy responses. We compared the relative weight of importation (due to air travel) and on-site amplification (due to the characteristics of each area: demographics, local NPIs, public health). Which of these types of factors affected the precocity and the severity of the COVID-19 pandemic during its first global wave in April–October 2020?

Our analyses inquired whether the timing and strength of the COVID-related death toll was systematically linked to the size of passenger flows and the connectedness of sub-national areas through airline routes during the first pandemic wave. We found this to be only partially the case.

Regarding the *precocity* of the emergence of COVID-19 as a cause of death, airport centrality is not significant and the volume of air passengers is found to be non-significant once demographic and public health characteristics of local areas are controlled for. This finding diverges with most of the simulation-based literature—possibly also because we focus on deaths rather than cases (i.e., the spread of the virus). Results are somewhat less straightforward when it comes to the relationship between air travel and the *severity* of the COVID-19 death numbers, which is also more controversial in the literature. On the one hand, the absence of airports or full travel bans systematically lead to a lower mortality *ceteris paribus*, showing that air travel does have a bearing on the severity of the pandemic. Moreover, the number of incoming passengers is also associated with higher levels of COVID-19 deaths. On the other hand, we find that this effect declines as the pandemic progresses and that the network centrality of airports is at no point a significant predictor of COVID-related mortality. Area-specific factors are found to be directly impactful, as well as an early onset of the pandemic, which leads to a higher death toll across the board. In this regard, we rejoin the literature emphasizing the overarching importance of local NPIs to contain transmission of the virus.

Policy-wise, further insights emerged when we dug deeper into the impact of air travel restrictions. Such measures are particularly debated, not the least because they challenge the International Health Regulations (IHR), a pact on collective action, which says that health measures implemented by countries ‘shall not be more restrictive of international traffic and not more invasive or intrusive to persons than reasonably available alternatives’^54^. Our results suggest that more drastic travel limitation policies were also more effective in cutting COVID-19 mortality. Regions that adopted milder travel restrictions have experienced a higher number of COVID-related deaths. Retrospectively, the policy lesson to be taken is that full bans paid off more than alternative air travel limitation measures particularly in the early stages of the pandemic, regardless of all other local conditions. Their effectiveness, however, appears to have diminished over time much like other NPIs more generally^55^.

Our analyses have a number of limitations that we acknowledge. First, our units are sub-national areas of different size, in terms of population and surface. Second, our main dependent variable—the weekly incidence of COVID-19 deaths—is far from perfect, as the imputation of deaths to the virus was not harmonized with a standard protocol in all of the areas we covered, especially in cases of comorbidity and in the early stages of the pandemic^56^. The future use of excess deaths may improve estimations, as underreporting of COVID-19 mortality was widespread to varying degrees in different countries; unfortunately, such measure is currently available at the national level only^57^. Statistics about COVID-related hospitalizations—still unavailable as we write on a subnational scale—would be equally illuminating. Some of our independent variables suffer from limitations as well. While very detailed, data about air passengers account for no more than a part of long-haul human mobility. People also travel by train, bus, car and boats. Moreover, in absence of more granular information, we impute national data about comorbidities to our subnational areas, assuming little variations country-wide, which may not always be the case. This may inflate error terms in our estimates. Another evident limit relates to time: our analysis is restricted to the first wave of the pandemic. Future research should also seek to expand the geographical scope of the analysis. Although we have an unprecedented coverage of areas hit by the virus, they are mostly in developed parts of the world. This may have led us to underestimate other potential local conditions boosting the severity of the disease (notably, poverty)^58^.

In conclusion, our results suggest that the impact of long-distance mobility approximates a ‘travel as spark’ principle, whereby the absence of air travel reduces epidemic risk drastically. However, once some travel occurs, its impact on the severity of the pandemic is only in part associated with the volume of incoming passengers, and not at all with the position of airports in the global network of airline connections. This is possibly because once the infection arrives in an area, the harm it causes is largely driven by within-area determinants of further spread. In this sense, local transmission risk outweighs the risk of virus importation.

## Supplementary Information

Supplementary Information.

## Data Availability

The dataset created for this article is publicly available in the Harvard Dataverse repository: https://doi.org/10.7910/DVN/SEGOVA.

## Code availability

The code implementing the model is available from the corresponding author on reasonable request.
