# Supplementary material for: The impact of air travel on the precocity and severity of COVID-19 deaths in sub-national areas across 45 countries
Source: Sci Rep. 2022 Oct 3;12:16522. doi: 10.1038/s41598-022-20263-y (PMC9527720; doi:10.1038/s41598-022-20263-y)
Supplement: Supplementary file 1 — Supplementary Information. [file 41598_2022_20263_MOESM1_ESM.docx]

**Supplementary information**

**Tables and Figures A (Sample description)**

Table A1. Descriptive statistics

|  | **Average** | | **April** | | **May** | | **June** | | **July** | | **August** | | **September** | | **October** | |
| --- | --- | --- | --- | --- | --- | --- | --- | --- | --- | --- | --- | --- | --- | --- | --- | --- |
|  | Mean | SD | Mean | SD | Mean | SD | Mean | SD | Mean | SD | Mean | SD | Mean | SD | Mean | SD |
| **Dependent variable** |  |  |  |  |  |  |  |  |  |  |  |  |  |  |  |  |
| Weekly COVID-19 deaths | 51 | 185 | 64 | 269 | 62 | 199 | 39 | 112 | 39 | 112 | 51 | 164 | 44 | 144 | 43 | 141 |
| **International mobility** |  |  |  |  |  |  |  |  |  |  |  |  |  |  |  |  |
| Passengers (lag 1 month) | 113323 | 259198 | 185294 | 387729 | 55113 | 122884 | 68949 | 156910 | 82709 | 195028 | 133716 | 276556 | 126904 | 266989 | 139919 | 292615 |
| Importation risk (lag 1 month) | 1922 | 2260 | 601 | 701 | 1545 | 1365 | 1900 | 2596 | 2044 | 2747 | 2104 | 2496 | 2571 | 2321 | 2501 | 2072 |
| Airport centrality (lag 1 month) | 679 | 5068 | 4165 | 13446 | 202 | 1077 | 128 | 577 | 71 | 302 | 271 | 1272 | 121 | 644 | 162 | 649 |
| **Travel bans (%)** |  |  |  |  |  |  |  |  |  |  |  |  |  |  |  |  |
| Total ban | 34 | 47 | 7 | 25 | 55 | 50 | 54 | 50 | 31 | 46 | 34 | 48 | 21 | 41 | 23 | 42 |
| No or minimal restrictions | 8 | 28 | 38 | 49 | 3 | 16 | 7 | 26 | 10 | 30 | 8 | 27 | 8 | 27 | 8 | 27 |
| Partial ban | 58 | 49 | 55 | 50 | 42 | 49 | 39 | 49 | 59 | 49 | 58 | 49 | 71 | 45 | 69 | 46 |
| **Local factors** |  |  |  |  |  |  |  |  |  |  |  |  |  |  |  |  |
| Population (thousands) | 5510 | 14872 | 5510 | 14872 | 5510 | 14872 | 5510 | 14872 | 5510 | 14872 | 5510 | 14872 | 5510 | 14872 | 5510 | 14872 |
| Oxford stringency index (lag 3 weeks) | 63 | 20 | 23 | 19 | 83 | 11 | 76 | 11 | 63 | 16 | 59 | 16 | 59 | 16 | 54 | 17 |
| Real GDP pc PPP | 31946 | 21690 | 31946 | 21713 | 31946 | 21713 | 31946 | 21713 | 31946 | 21713 | 31946 | 21713 | 31946 | 21713 | 31946 | 21713 |
| Population density (pop/km2) | 336 | 1234 | 336 | 1236 | 336 | 1236 | 336 | 1236 | 336 | 1236 | 336 | 1236 | 336 | 1236 | 336 | 1236 |
| Hospital beds per 1000 | 4 | 3 | 4 | 3 | 4 | 3 | 4 | 3 | 4 | 3 | 4 | 3 | 4 | 3 | 4 | 3 |
| Share of 65+ (%) | 16 | 6 | 16 | 6 | 16 | 6 | 16 | 6 | 16 | 6 | 16 | 6 | 16 | 6 | 16 | 6 |
| Cardiovascular deaths (x 100k) | 207 | 123 | 207 | 123 | 207 | 123 | 207 | 123 | 207 | 123 | 207 | 123 | 207 | 123 | 207 | 123 |
| Cancer deaths (x 100k) | 121 | 20 | 121 | 20 | 121 | 20 | 121 | 20 | 121 | 20 | 121 | 20 | 121 | 20 | 121 | 20 |
| Prevalence of adult obesity (%) | 23 | 8 | 23 | 8 | 23 | 8 | 23 | 8 | 23 | 8 | 23 | 8 | 23 | 8 | 23 | 8 |
| Week of first COVID death | 13 | 3 | 13 | 3 | 13 | 3 | 13 | 3 | 13 | 3 | 13 | 3 | 13 | 3 | 13 | 3 |
| Observations | 14570 | | 470 | | 470 | | 470 | | 470 | | 470 | | 470 | | 470 | |

Source: *Sub-National COVID-19 Incidence and Determinants Dataset.*

**Tables and Figures B (Precocity)**

Table B1. The impact of late 2019 air passenger traffic on the precocity of COVID-19 outbreaks (occurrence of 1^st^ and 10^th^ death) *including outlier regions* (India, Japan regions and US regions). OLS regressions without and with continent fixed effects

B1A. Airline passengers from Chinese airports

| DV: Calendar week | (1) | (2) | (3) | (4) | (5) | (6) | (7) | (8) |
| --- | --- | --- | --- | --- | --- | --- | --- | --- |
| of 1^st^/10^th^ COVID-19 death | Wk 1st | Wk 1st | Wk 1st | Wk 1st | Wk 10th | Wk 10th | Wk 10th | Wk 10th |
| No airport in region | .239 | .0616 | .548 | .162 | 1.810^*^ | 1.191 | 1.931^**^ | 1.350^*^ |
|  | (.400) | (.398) | (.351) | (.332) | (.808) | (.770) | (.691) | (.668) |
| Inbound passengers (end 2019) | -.803^***^ | -.551^***^ | -.582^***^ | -.445^***^ | -1.569^***^ | -.667^*^ | -.702^**^ | -.479 |
|  | (.137) | (.151) | (.136) | (.129) | (.272) | (.292) | (.267) | (.258) |
| Airport centrality (end 2019) | -.196 | -.190 | -.190 | -.076 | -.031 | -.061 | -.172 | -.334 |
|  | (.135) | (.146) | (.129) | (.123) | (.267) | (.275) | (.247) | (.242) |
| Population |  | -.273^*^ | -.509^***^ | -.716^***^ |  | -.544^*^ | -.925^***^ | -1.439^***^ |
|  |  | (.138) | (.124) | (.131) |  | (.263) | (.238) | (.258) |
| Population density (pop/km2) |  | -.0120 | -.0512 | -.249^*^ |  | .0542 | -.094 | -.388 |
|  |  | (.143) | (.125) | (.121) |  | (.270) | (.240) | (.237) |
| Real GDP pc PPP |  | -.558^***^ | .0229 | .257 |  | -2.307^***^ | -.623^*^ | -.337 |
|  |  | (.142) | (.137) | (.136) |  | (.305) | (.313) | (.323) |
| Hospital beds per 1000 residents |  |  |  | -.277 |  |  |  | .808^*^ |
|  |  |  |  | (.184) |  |  |  | (.366) |
| Share of 65+ |  |  |  | -.815^***^ |  |  |  | -1.706^***^ |
|  |  |  |  | (.192) |  |  |  | (.394) |
| Cardiovascular death rate |  |  |  | 1.025^***^ |  |  |  | .308 |
|  |  |  |  | (.247) |  |  |  | (.512) |
| Cancer death rate |  |  |  | .441^*^ |  |  |  | 1.418^***^ |
|  |  |  |  | (.181) |  |  |  | (.364) |
| Prevalence of adult obesity |  |  |  | -.754^**^ |  |  |  | -2.906^***^ |
|  |  |  |  | (.271) |  |  |  | (.543) |
| Observations | 489 | 489 | 489 | 489 | 465 | 465 | 465 | 465 |
| R-squared | .091 | .121 | .341 | .438 | .095 | .196 | .376 | .445 |
| Continent Fixed Effects | No | No | Yes | Yes | No | No | Yes | Yes |

B1B. Airline passengers from non-Chinese airports

| DV: Calendar week | (1) | (2) | (3) | (4) | (5) | (6) | (7) | (8) |
| --- | --- | --- | --- | --- | --- | --- | --- | --- |
| of 1^st^/10^th^ COVID-19 death | Wk 1st | Wk 1st | Wk 1st | Wk 1st | Wk 10th | Wk 10th | Wk 10th | Wk 10th |
| No airport in region | .567 | .200 | .677 | .243 | 2.506^**^ | 1.345 | 2.076^**^ | 1.483^*^ |
|  | (.405) | (.399) | (.349) | (.334) | (.823) | (.771) | (.691) | (.664) |
| Inbound passengers (end 2019) | -.540^***^ | -.409^*^ | -.664^***^ | -.340^*^ | -.635^*^ | -.207 | -.685^*^ | -.827^**^ |
|  | (.162) | (.162) | (.142) | (.145) | (.322) | (.306) | (.277) | (.285) |
| Airport centrality (end 2019) | -.115 | -.0540 | .0829 | -.001 | -.114 | -.069 | .089 | -.004 |
|  | (.162) | (.175) | (.152) | (.143) | (.323) | (.329) | (.294) | (.278) |
| Population |  | -.385^**^ | -.609^***^ | -.828^***^ |  | -.720^**^ | -1.058^***^ | -1.490^***^ |
|  |  | (.133) | (.117) | (.125) |  | (.252) | (.227) | (.243) |
| Population density (pop/km2) |  | -.095 | -.178 | -.320^**^ |  | .001 | -.227 | -.499^*^ |
|  |  | (.145) | (.126) | (.121) |  | (.274) | (.243) | (.237) |
| Real GDP pc PPP |  | -.700^***^ | -.045 | .186 |  | -2.563^***^ | -.749^*^ | -.413 |
|  |  | (.135) | (.132) | (.135) |  | (.283) | (.302) | (.314) |
| Hospital beds per 1000 residents |  |  |  | -.168 |  |  |  | 1.085^**^ |
|  |  |  |  | (.192) |  |  |  | (.376) |
| Share of 65+ |  |  |  | -.805^***^ |  |  |  | -1.668^***^ |
|  |  |  |  | (.193) |  |  |  | (.392) |
| Cardiovascular death rate |  |  |  | 1.007^***^ |  |  |  | .120 |
|  |  |  |  | (.252) |  |  |  | (.516) |
| Cancer death rate |  |  |  | .433^*^ |  |  |  | 1.446^***^ |
|  |  |  |  | (.182) |  |  |  | (.363) |
| Prevalence of adult obesity |  |  |  | -.930^***^ |  |  |  | -3.155^***^ |
|  |  |  |  | (.270) |  |  |  | (.537) |
| Observations | 489 | 489 | 489 | 489 | 465 | 465 | 465 | 465 |
| R-squared | .048 | .108 | .345 | .430 | .037 | .187 | .375 | .451 |
| Continent Fixed Effects | No | No | Yes | Yes | No | No | Yes | Yes |

Standard errors in parentheses. ^*^ *p* < .05, ^**^ *p* < .01, ^***^ *p* < .001.

Source: *Sub-National COVID-19 Incidence and Determinants Dataset.*

Figure B1. Scatterplot of the precocity of COVID-19 outbreak (occurrence of 1^st^ death) and incoming passengers from China or from other airports


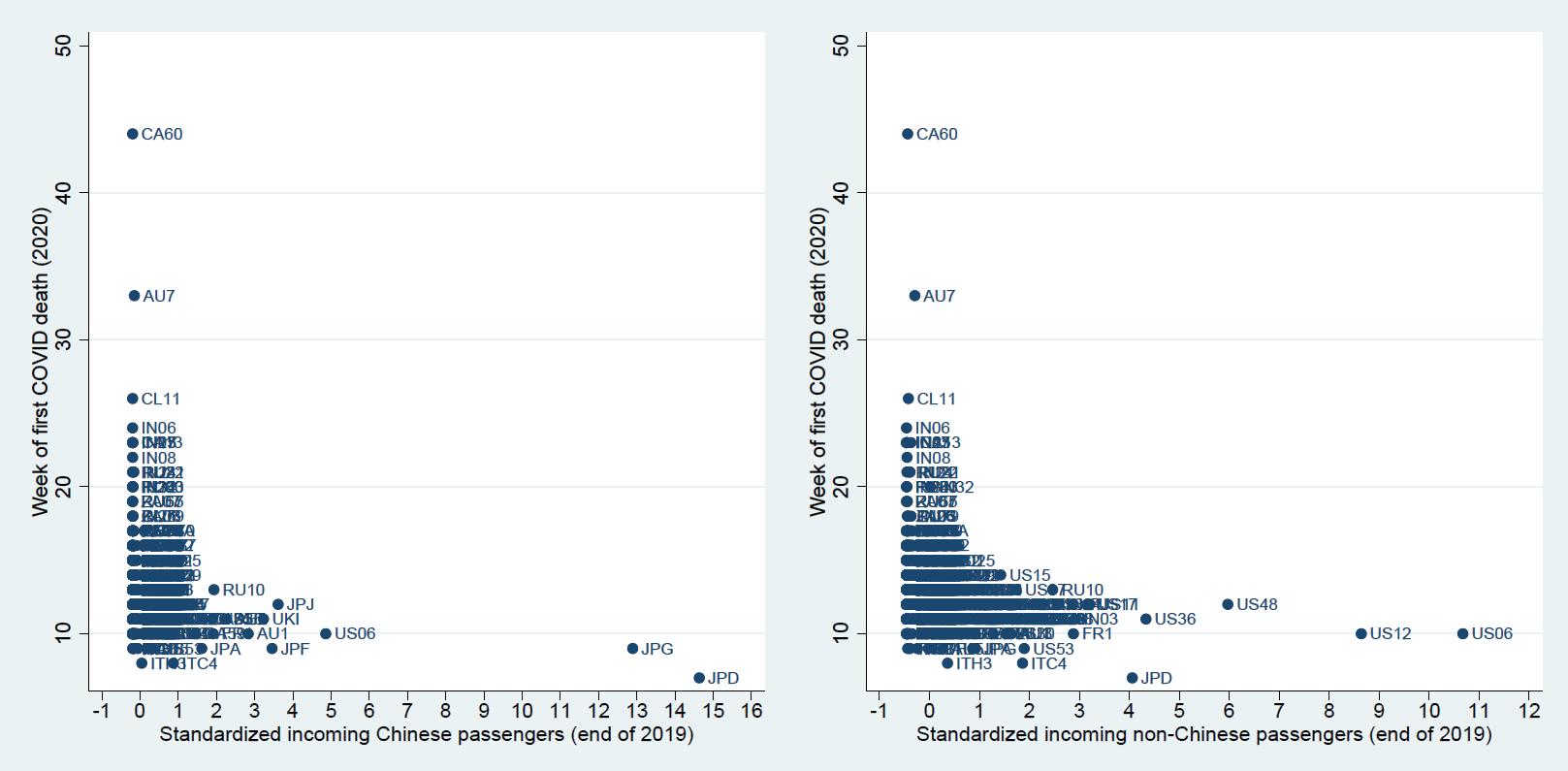


Source: *Sub-National COVID-19 Incidence and Determinants Dataset.*

Figure B2. The impact of air travel on the precocity of first COVID-19 death: measuring robustness to the exclusion of countries


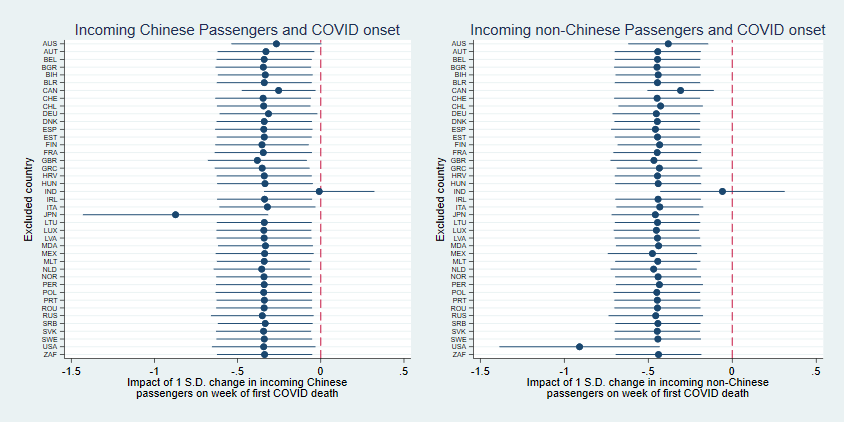


Note: outcomes control for the covariates of models 4 in Tables B1A and B1B.

Source: *Sub-National COVID-19 Incidence and Determinants Dataset.*

Table B2. The impact of late 2019 air passenger traffic on the precocity of COVID-19 outbreaks (occurrence of 1^st^ and 10^th^ *case*) *excluding outlier regions* (India, Japan regions and US regions). OLS regressions with and without continent fixed effects

B2A. Airline passengers from Chinese airports

| DV: Calendar week | (1) | (2) | (3) | (4) | (5) | (6) | (7) | (8) |
| --- | --- | --- | --- | --- | --- | --- | --- | --- |
| of 1^st^/10^th^ COVID-19 case | Wk 1st | Wk 1^st^ | Wk 1st | Wk 1^st^ | Wk 10th | Wk 10th | Wk 10th | Wk 10th |
| No airport in region | .851^*^ | .533 | .776^*^ | .349 | .419 | -.00146 | .474 | .114 |
|  | (.338) | (.330) | (.321) | (.300) | (.335) | (.321) | (.291) | (.277) |
| Inbound passengers (end 2019) | -1.982^***^ | -1.040^***^ | -1.029^***^ | -.766^**^ | -1.098^***^ | .0393 | -.160 | .0580 |
|  | (.231) | (.282) | (.286) | (.268) | (.227) | (.273) | (.258) | (.247) |
| Airport centrality (end 2019) | .0570 | .0842 | .214 | .142 | -.452^*^ | -.426^*^ | -.132 | -.206 |
|  | (.213) | (.222) | (.215) | (.199) | (.209) | (.214) | (.194) | (.182) |
| Population |  | -2.356^***^ | -2.481^***^ | -2.297^***^ |  | -2.830^***^ | -2.884^***^ | -2.783^***^ |
|  |  | (.512) | (.532) | (.494) |  | (.499) | (.485) | (.459) |
| Population density (pop/km2) |  | -.0881 | -.0855 | -.214 |  | -.0782 | -.0317 | -.145 |
|  |  | (.176) | (.169) | (.157) |  | (.170) | (.152) | (.144) |
| Real GDP pc PPP |  | -.429^***^ | -.182 | -.0390 |  | -.540^***^ | -.225^*^ | -.093 |
|  |  | (.115) | (.121) | (.118) |  | (.111) | (.109) | (.109) |
| Hospital beds per 1000 residents |  |  |  | .0349 |  |  |  | .088 |
|  |  |  |  | (.226) |  |  |  | (.207) |
| Share of 65+ |  |  |  | -.895^***^ |  |  |  | -.757^***^ |
|  |  |  |  | (.168) |  |  |  | (.156) |
| Cardiovascular death rate |  |  |  | .964^***^ |  |  |  | .776^***^ |
|  |  |  |  | (.225) |  |  |  | (.207) |
| Cancer death rate |  |  |  | .446^**^ |  |  |  | .303^*^ |
|  |  |  |  | (.166) |  |  |  | (.153) |
| Prevalence of adult obesity |  |  |  | -.826^**^ |  |  |  | -.709^*^ |
|  |  |  |  | (.318) |  |  |  | (.293) |
| Observations | 457 | 457 | 457 | 457 | 455 | 455 | 455 | 455 |
| R-squared | .164 | .228 | .308 | .425 | .0792 | .186 | .365 | .452 |
| Continent Fixed Effects | No | No | Yes | Yes | No | No | Yes | Yes |

B2B. Airline passengers from non-Chinese airports

| DV: Calendar week | (1) | (2) | (3) | (4) | (5) | (6) | (7) | (8) |
| --- | --- | --- | --- | --- | --- | --- | --- | --- |
| of 1^st^/10^th^ COVID-19 case | Wk 1st | Wk 1st | Wk 1st | Wk 1st | Wk 10th | Wk 10th | Wk 10th | Wk 10th |
| No airport in region | .481 | .394 | .629 | .269 | .121 | -.0287 | .409 | .106 |
|  | (.338) | (.331) | (.320) | (.298) | (.330) | (.320) | (.287) | (.274) |
| Inbound passengers (end 2019) | -1.483^***^ | -.663^**^ | -.571^*^ | -.578^**^ | -1.175^***^ | -.270 | -.278 | -.209 |
|  | (.166) | (.228) | (.221) | (.212) | (.161) | (.220) | (.198) | (.195) |
| Airport centrality (end 2019) | -.005 | .128 | .156 | .175 | -.093 | .043 | .102 | .093 |
|  | (.128) | (.133) | (.130) | (.121) | (.124) | (.128) | (.116) | (.110) |
| Population |  | -3.002^***^ | -3.508^***^ | -2.551^***^ |  | -2.932^***^ | -3.427^***^ | -2.955^***^ |
|  |  | (.623) | (.614) | (.606) |  | (.606) | (.555) | (.565) |
| Population density (pop/ km2) |  | -.060 | -.044 | -.180 |  | -.122 | -.028 | -.151 |
|  |  | (.174) | (.167) | (.155) |  | (.167) | (.149) | (.142) |
| Real GDP pc PPP |  | -.355^**^ | -.155 | .003 |  | -.517^***^ | -.208 | -.085 |
|  |  | (.124) | (.127) | (.122) |  | (.119) | (.113) | (.112) |
| Hospital beds per 1000 residents |  |  |  | .0366 |  |  |  | .102 |
|  |  |  |  | (.225) |  |  |  | (.206) |
| Share of 65+ |  |  |  | -.907^***^ |  |  |  | -.740^***^ |
|  |  |  |  | (.168) |  |  |  | (.155) |
| Cardiovascular death rate |  |  |  | .926^***^ |  |  |  | .719^***^ |
|  |  |  |  | (.225) |  |  |  | (.206) |
| Cancer death rate |  |  |  | .410^*^ |  |  |  | .316^*^ |
|  |  |  |  | (.165) |  |  |  | (.152) |
| Prevalence of adult obesity |  |  |  | -.580 |  |  |  | -.695^*^ |
|  |  |  |  | (.322) |  |  |  | (.296) |
| Observations | 456 | 456 | 456 | 456 | 454 | 454 | 454 | 454 |
| R-squared | .191 | .237 | .316 | .435 | .140 | .203 | .385 | .466 |
| Continent Fixed Effects | No | No | Yes | Yes | No | No | Yes | Yes |

Standard errors in parentheses. ^*^ *p* < .05, ^**^ *p* < .01, ^***^ *p* < .001.

Source: *Sub-National COVID-19 Incidence and Determinants Dataset.*

**Tables and Figures C (severity)**

Table C1. The impact of air passenger traffic on the severity of COVID-19 deaths in April-October 2020, controlling for population mixing (NPI), structural predispositions and recursive effects. Generalized linear mixed-effects models with sequential addition of covariates

| DV: Weekly COVID-19 deaths | Model 0 | Model 1 | Model 2 | Model 3 | Model 4 | Model 5 | Model 6 |
| --- | --- | --- | --- | --- | --- | --- | --- |
| Partial travel ban (lag 3 weeks)° | 1.014* [1.002;1.025] | 1.013* [1.002;1.025] | 1.013* [1.002;1.024] | 1.013* [1.001;1.024] | 1.013* [1.001;1.024] | 1.001 [.989;1.012] | .957*** [.946;.969] |
| None/Minimal travel restrictions (lag 3 weeks)° | 1.058*** [1.049;1.067] | 1.058*** [1.049;1.067] | 1.058*** [1.049;1.067] | 1.057*** [1.049;1.066] | 1.057*** [1.048;1.066] | 1.021*** [1.012;1.03] | .992 [.983;1.001] |
| Calendar week° | .984 [.885;1.093] | .980 [.931;1.031] | .979 [.932;1.029] | .968 [.926;1.012] | .971 [.927;1.016] | .988 [.96;1.016] | 1.018 [.991;1.046] |
| Population size |  | 2.084*** [1.804;2.408] | 2.123*** [1.839;2.45] | 1.471*** [1.277;1.694] | 1.457*** [1.267;1.675] | 1.479*** [1.284;1.704] | 1.477*** [1.283;1.702] |
| Population density (pop/km2) |  |  | 1.180** [1.043;1.335] | 1.026 [.918;1.147] | 1.028 [.921;1.148] | 1.041 [.93;1.164] | 1.042 [.932;1.165] |
| Share of 65+ |  |  | 1.539** [1.162;2.038] | 1.245 [.969;1.6] | 1.271 [.991;1.63] | 1.172 [.909;1.511] | 1.169 [.908;1.505] |
| Real GDP pc PPP |  |  | 1.066 [.908;1.251] | 1.149 [.997;1.323] | 1.123 [.975;1.293] | 1.099 [.952;1.269] | 1.092 [.947;1.26] |
| Hospital beds per 1000 residents |  |  | .786 [.399;1.547] | .703 [.393;1.26] | .697 [.387;1.253] | .656 [.347;1.241] | .614 [.335;1.124] |
| Cardiovascular death rate |  |  | .853 [.46;1.583] | 1.189 [.694;2.037] | 1.251 [.727;2.152] | 1.494 [.832;2.682] | 1.503 [.859;2.629] |
| Cancer death rate |  |  | .839 [.459;1.534] | .791 [.473;1.322] | .81 [.482;1.36] | .782 [.447;1.37] | .799 [.469;1.362] |
| Prevalence of adult obesity |  |  | 1.668 [.769;3.621] | 1.62 [.842;3.117] | 1.574 [.813;3.049] | 1.453 [.708;2.983] | 1.527 [.774;3.011] |
| Week of first COVID death |  |  |  | .679*** [.636;.726] | .686*** [.642;.733] | .693*** [.648;.742] | .693*** [.648;.740] |
| No airport in region |  |  |  |  | .612** [.443;.845] | .772 [.555;1.074] | .773 [.557;1.074] |
| Centrality (lag 1 mnt)° |  |  |  |  |  | .980*** [.975;.986] | .974*** [.969;.980] |
| Inbound passengers (lag 1 mnt)° |  |  |  |  |  | 1.016*** [1.011;1.021] | 1.009*** [1.004;1.014] |
| Importation risk (lag 1 mnt)° |  |  |  |  |  | .981*** [.978;.984] | .981*** [.977;.984] |
| Importation risk x inbound passengers (lag 1 mnt)° |  |  |  |  |  | 1.067* [1.012;1.125] | 1.054* [1.000;1.111] |
| Oxford stringency index (lag 3 wks)° |  |  |  |  |  |  | .974*** [.970;.979] |
|  |  |  |  |  |  |  |  |
| NUTS in (Country in Continent)-Intercept | 1.4e+00 | 1.4e+00 | 1.3e+00 | 1.3e+00 | 1.5e+00 | 1.5e+00 | 1.6e+00 |
| NUTS in (Country in Continent)-Slope | 5.4e-02 | 5.5e-02 | 5.2e-02 | 5.2e-02 | 5.2e-02 | 5.2e-02 | 5.2e-02 |
| NUTS in (Country in Continent)-Corr | -6.5e-01 | -6.5e-01 | -6.2e-01 | -6.2e-01 | -6.1e-01 | -6.2e-01 | -5.7e-01 |
| Country in Continent -Intercept | 1.3e+00 | 1.4e+00 | 1.3e+00 | 1.3e+00 | 1.5e+00 | 1.7e+00 | 1.7e+00 |
| Country in Continent -Slope | 7.9e-02 | 8.5e-02 | 1.1e-01 | 1.1e-01 | 1.1e-01 | 1.1e-01 | 1.1e-01 |
| Country in Continent -Corr | 4.0e-02 | -1.0e-01 | -1.1e-01 | -1.3e-01 | -2.0e-01 | -3.3e-01 | -3.9e-01 |
| Continent -Intercept | .0e+00 | 2.1e-04 | 1.2e-01 | 9.4e-02 | 3.6e-01 | 5.8e-01 | 2.8e-04 |
| Continent -Slope | 4.2e-07 | 1.1e-06 | 2.2e-02 | 2.0e-02 | 2.9e-02 | 3.3e-02 | 1.1e-01 |
| Continent -Corr | NaN | -1.0e+00 | -1.0e+00 | -1.0e+00 | -1.0e+00 | -1.0e+00 | -1.5e-01 |

^*^ *p* < .05, ^**^ *p* < .01, ^***^ *p* < .001. ° by week.

Source: *Sub-National COVID-19 Incidence and Determinants Dataset.*

Figure C1. Conditional modes of the random effects (intercept and slope) from the negative binomial generalized linear mixed model of Table C1 (model 6)


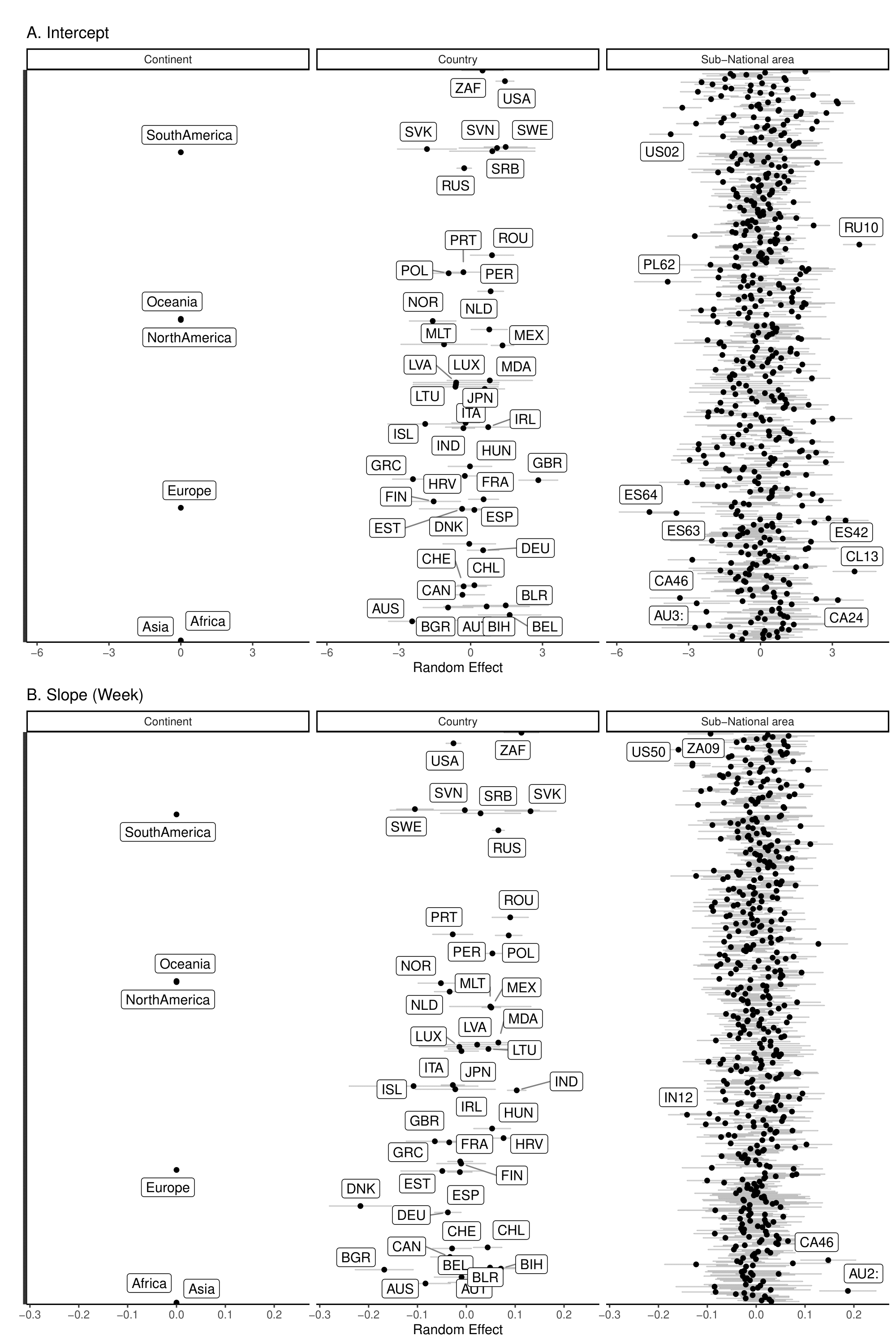


Source: *Sub-National COVID-19 Incidence and Determinants Dataset.*

Table C2. The impact of air passenger traffic on the severity of COVID-19 deaths in April-October 2020, controlling for population mixing (NPI), structural predispositions and recursive effects. Negative binomial regressions with continent fixed effects

| DV: Weekly COVID-19 deaths | (1) | (2) | (3) | (4) | (5) | (6) | (7) |
| --- | --- | --- | --- | --- | --- | --- | --- |
|  | April | May | June | July | Aug | Sep | Oct |
| *International mobility* |  |  |  |  |  |  |  |
| No airport in region | .607^*^ | .491^***^ | .275^***^ | .582^*^ | .793 | .897 | .654^*^ |
|  | [.385,.956] | [.325,.741] | [.166,.454] | [.365,.926] | [.508,1.240] | [.574,1.404] | [.437,.980] |
| Inbound passengers (lag 1 mnt) | 1.387^***^ | 1.669^***^ | 2.006^**^ | 1.495^***^ | 1.588^***^ | 1.425^***^ | 1.315^***^ |
|  | [1.149,1.675] | [1.372,2.030] | [1.267,3.177] | [1.193,1.875] | [1.282,1.967] | [1.197,1.697] | [1.118,1.546] |
| Importation risk (lag 1 mnt) | 1.180 | 1.147 | .978 | .731^*^ | 1.153 | 1.339^**^ | 1.163 |
|  | [.995,1.399] | [.972,1.353] | [.651,1.468] | [.576,.928] | [.940,1.414] | [1.120,1.600] | [.995,1.358] |
| Importation risk x inbound passengers | 1.303^*^ | 1.413^**^ | 1.801 | .833 | .935 | 1.021 | 1.131 |
|  | [1.034,1.643] | [1.141,1.751] | [.797,4.070] | [.582,1.192] | [.815,1.072] | [.880,1.185] | [.910,1.405] |
| Airport centrality (lag 1 mnt) | .881 | .895 | .954 | 1.018 | .955 | 1.159 | 1.142 |
|  | [.767,1.011] | [.786,1.018] | [.796,1.145] | [.863,1.199] | [.792,1.153] | [.942,1.427] | [.982,1.328] |
| *Travel restrictions (ref: total travel ban)* |  |  |  |  |  |  |  |
| None/minimal (lag 3 wks) | 4.611^***^ | 4.746^***^ | 3.108^***^ | 2.953^***^ | 4.495^***^ | 2.543 | .900 |
|  | [2.725,7.804] | [2.284,9.859] | [1.607,6.012] | [1.560,5.591] | [1.921,1.52] | [.598,1.81] | [.401,2.020] |
| Partial ban (lag 3 wks) | 4.716^***^ | .599^**^ | 2.029^***^ | 2.024^**^ | 1.329 | 1.285 | .569 |
|  | [2.616,8.502] | [.429,.838] | [1.376,2.992] | [1.258,3.255] | [.623,2.835] | [.364,4.537] | [.291,1.111] |
| *Containment policies* |  |  |  |  |  |  |  |
| Oxford stringency index (lag 3 wks) | .821^*^ | 1.021 | 1.279^*^ | 2.152^***^ | 1.297 | 1.565^**^ | 1.565^**^ |
|  | [.681,.991] | [.877,1.188] | [1.037,1.578] | [1.669,2.773] | [.970,1.734] | [1.155,2.120] | [1.160,2.113] |
| *Structural variables* |  |  |  |  |  |  |  |
| Population size | 1.311^*^ | 1.442^***^ | 1.190 | 1.623^***^ | 1.383^**^ | 1.408^**^ | 1.366^**^ |
|  | [1.053,1.632] | [1.169,1.779] | [.893,1.586] | [1.236,2.131] | [1.083,1.767] | [1.101,1.801] | [1.089,1.714] |
| Real GDP pc PPP | 1.127 | 1.103 | .940 | .880 | .781^*^ | .697^***^ | .678^***^ |
|  | [.913,1.391] | [.919,1.325] | [.720,1.227] | [.732,1.058] | [.633,.964] | [.575,.844] | [.570,.807] |
| Population density (pop/km2) | .987 | .923 | 1.072 | 1.098 | 1.003 | 1.183 | 1.083 |
|  | [.848,1.149] | [.802,1.062] | [.891,1.288] | [.924,1.304] | [.847,1.186] | [.958,1.460] | [.934,1.256] |
| Hospital beds per 1000 | .547^***^ | .851 | .779 | .641^**^ | .784 | .796 | .956 |
|  | [.432,.691] | [.681,1.064] | [.599,1.014] | [.480,.854] | [.574,1.072] | [.553,1.147] | [.707,1.292] |
| Share of 65+ | 1.099 | 1.201 | .900 | .874 | .655^**^ | .861 | .729^*^ |
|  | [.801,1.509] | [.951,1.518] | [.670,1.210] | [.664,1.148] | [.492,.870] | [.657,1.129] | [.566,.939] |
| Cardiovascular death rate | 1.645^**^ | 1.551^**^ | 3.302^***^ | 5.365^***^ | 3.179^***^ | 2.435^***^ | 1.622^**^ |
|  | [1.127,2.403] | [1.155,2.083] | [2.184,4.991] | [3.771,7.631] | [2.235,4.521] | [1.685,3.517] | [1.126,2.337] |
| Cancer death rate | 1.146 | .878 | .729^*^ | .642^***^ | .863 | 1.094 | 1.056 |
|  | [.908,1.448] | [.722,1.067] | [.565,.940] | [.508,.812] | [.667,1.116] | [.852,1.405] | [.845,1.321] |
| Prevalence of adult obesity | 1.952^***^ | 1.742^**^ | 1.236 | 2.541^***^ | .811 | .502^**^ | 1.259 |
|  | [1.344,2.834] | [1.189,2.551] | [.761,2.007] | [1.660,3.890] | [.535,1.228] | [.314,.802] | [.867,1.827] |
| *Recursive effects* |  |  |  |  |  |  |  |
| Week of first COVID death | .063^***^ | .155^***^ | .338^***^ | .456^***^ | .406^***^ | .471^***^ | .473^***^ |
|  | [.0419,.0962] | [.114,.210] | [.254,.448] | [.354,.588] | [.315,.523] | [.367,.603] | [.386,.579] |
| Lnalpha | 1.055 | 1.185^*^ | 1.840^***^ | 1.556^***^ | 1.471^***^ | 1.518^***^ | 1.204^**^ |
|  | [.902,1.233] | [1.035,1.357] | [1.600,2.115] | [1.339,1.809] | [1.267,1.707] | [1.313,1.754] | [1.047,1.383] |
| Avg. deaths per week | 63.84 | 62.19 | 38.56 | 38.85 | 5.81 | 44.45 | 43.03 |
|  |  |  |  |  |  |  |  |
| Observations | 470 | 470 | 470 | 470 | 470 | 470 | 470 |
| Pseudo R-squared | .210 | .145 | .108 | .129 | .134 | .118 | .110 |
| Continent Fixed Effects | Yes | Yes | Yes | Yes | Yes | Yes | Yes |

Standard errors in parentheses. ^*^ *p* < .05, ^**^ *p* < .01, ^***^ *p* < .001.

Source: *Sub-National COVID-19 Incidence and Determinants Dataset.*

Table C3. The impact of air passenger traffic on the severity of COVID-19 deaths in April-October 2020, controlling for population mixing (NPI), structural predispositions and recursive effects, *including air pollution*. Negative binomial regressions with continent fixed effects

| DV: Weekly COVID-19 deaths | (1) | (2) | (3) | (4) | (5) | (6) | (7) |
| --- | --- | --- | --- | --- | --- | --- | --- |
|  | April | May | June | July | Aug | Sep | Oct |
| *International mobility* |  |  |  |  |  |  |  |
| No airport in region | .726 | .482^**^ | .248^***^ | .448^**^ | .616 | 1.176 | .611^*^ |
|  | [.454,1.161] | [.306,.759] | [.139,.441] | [.269,.747] | [.370,1.026] | [.696,1.988] | [.386,.968] |
| Inbound passengers (lag 1 mnt) | 1.329^**^ | 1.792^***^ | 2.214^**^ | 1.579^***^ | 1.638^***^ | 1.513^***^ | 1.478^***^ |
|  | [1.098,1.608] | [1.450,2.215] | [1.335,3.671] | [1.233,2.024] | [1.267,2.117] | [1.217,1.881] | [1.222,1.788] |
| Importation risk (lag 1 mnt) | 1.166 | 1.160 | 1.005 | .682^**^ | 1.073 | 1.589^***^ | 1.142 |
|  | [.984,1.382] | [.964,1.395] | [.632,1.599] | [.514,.905] | [.848,1.356] | [1.271,1.987] | [.946,1.380] |
| Importation risk x inbound passengers | 1.356^*^ | 1.509^***^ | 1.629 | .699 | .896 | .975 | .987 |
|  | [1.064,1.729] | [1.199,1.899] | [.674,3.941] | [.456,1.072] | [.756,1.062] | [.829,1.147] | [.766,1.271] |
| Airport centrality (lag 1 mnt) | .929 | .865^*^ | .897 | 1.004 | .932 | 1.102 | 1.108 |
|  | [.800,1.078] | [.757,.989] | [.741,1.085] | [.837,1.204] | [.764,1.137] | [.884,1.373] | [.940,1.305] |
| *Travel restrictions (ref: total travel ban)* |  |  |  |  |  |  |  |
| None/minimal (lag 3 wks) | 4.025^***^ | 4.862^***^ | 3.334^**^ | 2.378^*^ | 17.17^***^ | 2.113 | .269^**^ |
|  | [2.236,7.245] | [1.974,11.98] | [1.348,8.245] | [1.122,5.043] | [5.257,56.09] | [.495,9.020] | [.110,.655] |
| Partial ban (lag 3 wks) | 2.763^**^ | .961 | 2.137^**^ | 2.404^**^ | 6.533^***^ | 2.946 | .579 |
|  | [1.474,5.179] | [.664,1.390] | [1.292,3.534] | [1.374,4.206] | [2.528,16.88] | [.915,9.486] | [.289,1.158] |
| *Containment policies* |  |  |  |  |  |  |  |
| Oxford stringency index (lag 3 wks) | .814^*^ | .822^*^ | 1.317 | 2.064^***^ | 1.256 | 1.570^**^ | 1.256 |
|  | [.673,.985] | [.681,.991] | [.959,1.809] | [1.494,2.851] | [.917,1.720] | [1.151,2.143] | [.918,1.717] |
| *Structural variables* |  |  |  |  |  |  |  |
| Population size | 1.364^**^ | 1.760^***^ | 1.157 | 1.599^**^ | 1.578^**^ | 1.349^*^ | 1.369^**^ |
|  | [1.095,1.701] | [1.382,2.241] | [.856,1.564] | [1.207,2.119] | [1.190,2.092] | [1.053,1.729] | [1.081,1.735] |
| Real GDP pc PPP | 1.265^*^ | 1.038 | .924 | .881 | .790 | .632^***^ | .638^***^ |
|  | [1.001,1.598] | [.859,1.255] | [.683,1.251] | [.717,1.082] | [.605,1.031] | [.507,.788] | [.525,.774] |
| Population density (pop/km2) | .824^*^ | .926 | 1.102 | 1.142 | 1.060 | 1.103 | 1.108 |
|  | [.688,.987] | [.780,1.098] | [.882,1.377] | [.933,1.399] | [.864,1.301] | [.887,1.372] | [.931,1.317] |
| Hospital beds per 1000 residents | .525^***^ | .763^*^ | .701^*^ | .511^***^ | .594^**^ | .651^*^ | .755 |
|  | [.406,.679] | [.590,.986] | [.514,.956] | [.347,.754] | [.416,.851] | [.437,.970] | [.538,1.061] |
| Share of 65+ | 1.125 | 1.166 | 1.045 | .920 | .775 | .772 | .645^**^ |
|  | [.791,1.600] | [.901,1.511] | [.731,1.492] | [.667,1.268] | [.534,1.123] | [.540,1.105] | [.478,.870] |
| Cardiovascular death rate | .745 | 1.269 | 5.783^***^ | 3.346^**^ | 3.939^***^ | 1.046 | .958 |
|  | [.400,1.389] | [.690,2.334] | [2.587,12.93] | [1.547,7.237] | [1.895,8.191] | [.528,2.073] | [.555,1.654] |
| Cancer death rate | 1.064 | .924 | .821 | .606^***^ | .845 | 1.277 | 1.084 |
|  | [.840,1.347] | [.759,1.124] | [.608,1.110] | [.471,.781] | [.630,1.134] | [.919,1.775] | [.832,1.413] |
| Prevalence of adult obesity | 5.443^***^ | 3.228^***^ | .791 | 4.963^***^ | 1.049 | .706 | 2.154^**^ |
|  | [3.144,9.422] | [1.734,6.009] | [.363,1.723] | [2.757,8.934] | [.536,2.052] | [.317,1.570] | [1.256,3.692] |
| Air pollution (PM 2.5) | 1.376^*^ | 1.163 | .927 | 1.006 | 1.019 | 1.286 | 1.159 |
|  | [1.031,1.837] | [.852,1.588] | [.670,1.283] | [.771,1.313] | [.773,1.343] | [.960,1.723] | [.912,1.473] |
| *Recursive effects* |  |  |  |  |  |  |  |
| Week of first COVID death | .058^***^ | .172^***^ | .360^***^ | .493^***^ | .487^***^ | .537^***^ | .547^***^ |
|  | [.0363,.0954] | [.116,.255] | [.257,.504] | [.368,.661] | [.361,.656] | [.402,.717] | [.429,.698] |
| Lnalpha | .904 | 1.053 | 1.908^***^ | 1.418^***^ | 1.435^***^ | 1.391^***^ | 1.133 |
|  | [.759,1.078] | [.908,1.221] | [1.636,2.225] | [1.198,1.679] | [1.215,1.694] | [1.180,1.638] | [.969,1.326] |
| Avg. deaths per week | 67.48 | 68.55 | 42.49 | 42.01 | 55.97 | 49.10 | 47.92 |
|  |  |  |  |  |  |  |  |
| Observations | 389 | 389 | 389 | 389 | 389 | 389 | 389 |
| Pseudo R-squared | .227 | .160 | .108 | .144 | .141 | .133 | .123 |
| Continent Fixed Effects | Yes | Yes | Yes | Yes | Yes | Yes | Yes |

Standard errors in parentheses. ^*^ *p* < .05, ^**^ *p* < .01, ^***^ *p* < .001.

Source: *Sub-National COVID-19 Incidence and Determinants Dataset.*

Table C4. The impact of air passenger traffic on the severity of COVID-19 deaths in the first 24 epidemiological weeks controlling for population mixing (NPI), structural predispositions and recursive effects. Negative binomial regressions with continent fixed effects

| DV: Weekly COVID-19 deaths | (1) | (2) | (3) | (4) | (5) | (6) | (7) |
| --- | --- | --- | --- | --- | --- | --- | --- |
|  | Epi wk 6 | Epi wk 10 | Epi wk 14 | Epi wk 18 | Epi wk 22 | Epi wk 26 | Epi wk 30 |
| *International mobility* |  |  |  |  |  |  |  |
| No airport in region | .406^***^ | .561^*^ | .782 | .791 | .400^***^ | .713 | .538^**^ |
|  | [.258,.640] | [.350,.900] | [.477,1.284] | [.483,1.297] | [.256,.624] | [.470,1.083] | [.353,.821] |
| Inbound passengers (lag 1 mnt) | 3.052^***^ | 2.682^***^ | 1.962^***^ | 1.530^**^ | 1.416^***^ | 1.401^***^ | 1.343^*^ |
|  | [2.267,4.110] | [1.900,3.786] | [1.417,2.717] | [1.174,1.993] | [1.165,1.721] | [1.168,1.679] | [1.066,1.691] |
| Importation risk (lag 1 mnt) | 1.171 | 1.237 | 1.222 | 1.111 | .962 | 1.060 | 1.043 |
|  | [.914,1.499] | [.923,1.657] | [.937,1.594] | [.893,1.384] | [.792,1.169] | [.897,1.251] | [.877,1.240] |
| Importation risk x inbound passengers | 1.701^*^ | 2.972^***^ | 2.058^**^ | .983 | 1.021 | .976 | .929 |
|  | [1.071,2.700] | [1.592,5.549] | [1.207,3.510] | [.709,1.363] | [.892,1.168] | [.849,1.122] | [.766,1.126] |
| Airport centrality (lag 1 mnt) | .679^***^ | .862 | 1.037 | 1.003 | 1.010 | 1.121 | 1.060 |
|  | [.556,.830] | [.708,1.050] | [.877,1.225] | [.832,1.209] | [.844,1.207] | [.901,1.395] | [.843,1.333] |
| *Travel restrictions (ref: total travel ban)* |  |  |  |  |  |  |  |
| None/minimal (lag 3 wks) | 2.750^**^ | 3.580^**^ | 3.535^***^ | 1.280 | 16.30^***^ | 2.931^**^ | 1.598 |
|  | [1.345,5.623] | [1.633,7.850] | [1.753,7.129] | [.663,2.471] | [5.107,52.03] | [1.401,6.133] | [.841,3.034] |
| Partial ban (lag 3 wks) | .996 | 1.408^*^ | 1.580^*^ | .971 | 3.706^*^ | 1.128 | .609 |
|  | [.691,1.436] | [1.003,1.975] | [1.008,2.477] | [.619,1.524] | [1.348,1.19] | [.582,2.186] | [.361,1.027] |
| *Containment policies* |  |  |  |  |  |  |  |
| Oxford stringency index (lag 3 wks) | 1.022 | 1.381^***^ | 2.181^***^ | 1.487^***^ | 1.384^*^ | 1.617^***^ | 1.174 |
|  | [.857,1.219] | [1.186,1.608] | [1.729,2.752] | [1.179,1.876] | [1.069,1.791] | [1.235,2.117] | [.855,1.612] |
| *Structural variables* |  |  |  |  |  |  |  |
| Population size | 1.018 | 1.257 | 1.203 | 1.307^*^ | 1.685^***^ | 1.581^***^ | 1.703^***^ |
|  | [.828,1.252] | [.984,1.607] | [.893,1.621] | [1.005,1.701] | [1.288,2.204] | [1.237,2.021] | [1.277,2.270] |
| Real GDP pc PPP | 1.076 | 1.031 | 1.026 | .742^**^ | .831 | .709^***^ | .684^***^ |
|  | [.867,1.335] | [.834,1.275] | [.808,1.301] | [.599,.920] | [.687,1.005] | [.607,.828] | [.583,.803] |
| Population density (pop/km2) | .961 | 1.027 | 1.087 | 1.134 | 1.025 | .996 | 1.238^*^ |
|  | [.811,1.138] | [.862,1.224] | [.894,1.323] | [.936,1.374] | [.877,1.198] | [.873,1.137] | [1.027,1.492] |
| Hospital beds per 1000 residents | .588^***^ | .958 | .985 | .506^***^ | .715^*^ | .906 | .801 |
|  | [.462,.747] | [.760,1.209] | [.764,1.270] | [.379,.675] | [.514,.994] | [.687,1.194] | [.591,1.086] |
| Share of 65+ | 1.058 | .945 | 1.141 | .974 | .652^**^ | .771^*^ | .605^***^ |
|  | [.795,1.408] | [.727,1.228] | [.860,1.515] | [.732,1.297] | [.493,.861] | [.599,.992] | [.465,.789] |
| Cardiovascular death rate | 1.121 | 1.342 | 4.854^***^ | 4.083^***^ | 2.499^***^ | 1.927^***^ | 1.313 |
|  | [.807,1.556] | [.981,1.835] | [3.142,7.498] | [2.909,5.730] | [1.786,3.497] | [1.392,2.668] | [.933,1.849] |
| Cancer death rate | .853 | .611^***^ | .774 | .849 | 1.422^**^ | 1.192 | 1.457^**^ |
|  | [.671,1.083] | [.492,.760] | [.598,1.000] | [.655,1.101] | [1.106,1.827] | [.948,1.499] | [1.122,1.892] |
| Prevalence of adult obesity | 1.294 | 1.991^***^ | .488^**^ | .847 | .401^***^ | .938 | 2.117^***^ |
|  | [.861,1.946] | [1.383,2.867] | [.308,.775] | [.566,1.268] | [.264,.609] | [.681,1.293] | [1.488,3.013] |
| *Recursive effects* |  |  |  |  |  |  |  |
| Week of first COVID death | .384^***^ | .392^***^ | .508^***^ | .509^***^ | .641^***^ | .601^***^ | .577^***^ |
|  | [.292,.504] | [.301,.512] | [.391,.660] | [.394,.657] | [.512,.804] | [.483,.747] | [.458,.728] |
| Lnalpha | 1.655^***^ | 1.576^***^ | 1.845^***^ | 1.736^***^ | 1.438^***^ | 1.352^***^ | 1.319^***^ |
|  | [1.454,1.884] | [1.378,1.802] | [1.599,2.128] | [1.495,2.015] | [1.243,1.664] | [1.169,1.565] | [1.150,1.513] |
| Avg. deaths per week | 91.65 | 51.27 | 42.74 | 47.56 | 5.57 | 44.48 | 55.98 |
|  |  |  |  |  |  |  |  |
| Observations | 478 | 478 | 476 | 470 | 469 | 460 | 434 |
| Pseudo R-squared | .112 | .0996 | .109 | .114 | .134 | .120 | .0870 |
| Continent Fixed Effects | Yes | Yes | Yes | Yes | Yes | Yes | Yes |

Standard errors in parentheses. ^*^ *p* < .05, ^**^ *p* < .01, ^***^ *p* < .001.

Source: *Sub-National COVID-19 Incidence and Determinants Dataset.*
